# A Review of Interactions between Insect Biological Control Agents and Semiochemicals

**DOI:** 10.3390/insects10120439

**Published:** 2019-12-05

**Authors:** Anamika Sharma, Ramandeep Kaur Sandhi, Gadi V. P. Reddy

**Affiliations:** 1 Montana State University, Western Triangle Agricultural Research Center, 9546 Old Shelby Rd, P.O. Box 656, Conrad, MT 59425, USA; anamika.sharma@montana.edu (A.S.); ramandeepkaursandhi@montana.edu (R.K.S.); 2 USDA-ARS, Southern Insect Management Research Unit, 141 Experiment Station Road, P.O. Box 346, Stoneville, MS 38776, USA

**Keywords:** pheromones, kairomones, IPM, entomopathogens, parasitoids, predators, microbial pesticides

## Abstract

Biological control agents and semiochemicals have become essential parts of the integrated pest management of insect pests over recent years, as the incorporation of semiochemicals with natural enemies and entomopathogenic microbials has gained significance. The potential of insect pheromones to attract natural enemies has mainly been established under laboratory conditions, while semiochemicals from plants have been used to attract and retain natural enemies in field conditions using strategies such as trap crops and the push–pull mechanism. The best-known semiochemicals are those used for parasitoids–insect pest–plant host systems. Semiochemicals can also aid in the successful dispersal of entomopathogenic microbials. The use of semiochemicals to disseminate microbial pathogens is still at the initial stage, especially for bacterial and viral entomopathogens. Future studies should focus on the integration of semiochemicals into management strategies for insects, for which several semiochemical compounds have already been studied. More effective formulations of microbial agents, such as granular formulations of entomopathogenic fungi (EPFs), along with bio-degradable trap materials, could improve this strategy. Furthermore, more studies to evaluate species-specific tactics may be needed, especially where more than one key pest is present.

## 1. Introduction

Biological management of insect pests is an integral part of integrated pest management (IPM) programs and requires active human involvement. Biological control strategies include classical (importation), inundative and inoculative augmentation, and conservation approaches [1]. Biological management of insect pests includes the use of predators [e.g., *Coccinella septempunctata* L. (Coleoptera: Coccinellidae)], parasitoids [e.g., *Trioxys complanatus* Quilis (Hymenoptera: Aphidiidae)], competitors [e.g., *Novomessor cockerelli* André and *Pogonomyrmex barbatus* Smith (both Hymenoptera: Formicidae)], and pathogens. Pathogens include bacteria [e.g., *Bacillus thuringiensis* Berliner (Bacillales: Bacillaceae)], fungi [e.g., *Beauveria bassiana* s.l. Bals.-Criv. Vuill. (Hypocreales: Cordycipitaceae)], viruses [Nuclear Polyhedrosis Virus, (Baculoviridae)], and nematodes [e.g., *Deladenus siricidicola* Bedding (Tylenchida: Neotylenchidae)] [2,3,4,5]. Conservation of the ecological systems is also an integral part of biological management. Furthermore, various biological control agents (BCA) can be used together to manage different insect pests [6].

Semiochemicals also play a major role in the biological management of arthropods, as they are involved in the interspecific chemical communications between plants, insect pests, parasitoids and predators and are being used in IPM [7,8,9]. Semiochemicals are organic compounds that act as signals and enable intra- (same species) and inter- (varied species) specific chemical communication [10]. Pheromones function between members of the same species, whereas other types of semiochemicals, known as allelochemicals, function between members of different species and include allomones (favorable to the emitter), kairomones (favorable to the receiver but not to the emitter), synomones (favorable to both the emitter and the receiver), and apneumones (emitted by an abiotic material favorable to the receiver and detrimental to an organism found in or on the abiotic material). Kasinger et al., and several other publications provide a detailed classification of the identified semiochemicals [11,12]. The semiochemicals belong to various chemical groups, including aldehydes, alcohols, sulfur-containing compounds, esters, terpenes, alkanes, heterocyclic aromatic compounds, proteins, amino acids, triglycerides, and salts. Due to the many types of semiochemicals, the interaction between insect pests, insect BCA and semiochemicals has several facets, which are gradually being explored. The interaction of semiochemicals with BCAs is an emerging field of study which has been gaining importance in recent years, as it has several benefits, including the establishment of a sustainable environment and a reduction in the use of conventional pesticides [12].

At present, pheromones and other semiochemicals are extensively used to manage agricultural, stored products, and forest insect pests using monitoring, mass trapping, mating disruption, attract-and-kill, and push–pull strategies. Several kairomones, allomones, and synomones are known today, and the names and details of these semiochemicals are available on websites such as Pherobase and Pherolist [13,14]. Interspecific semiochemicals can be used to attract natural enemies. The application of interspecific semiochemicals in the form of trap crops, and the incorporation of synthetic semiochemicals with the trap crops, creates a ‘push–pull’ mechanism, in which repellents and attractants are both used. Repellents “push” insects away from main crops and attractants “pull” them towards side or trap crops [8,13]. Synthetic repellents, non-host volatiles, host-derived semiochemicals, anti-aggregation pheromones, oviposition deterring pheromones, alarm pheromones, antifeedants and visual cues act as push components, whereas host volatiles, sex and aggregation pheromones, visual stimulants, and gustatory and oviposition stimulants act as pull components [13,14].

Semiochemicals are also implicated in attracting entomopathogenic microbials to manage insect pests [12,15] and can be used to disseminate the entomopathogenic microbials [16,17]. Auto-dissemination systems, which employee both entomopathogens and attractive, species-specific semiochemicals, enable a more effective pathogen dispersal. This system is also called ‘lure and kill’ or ‘attract and kill’ and is proving effective for the management of several insect pests [4,18]. Vega et al. [18] list examples of auto inoculators for the dispersal of microbials such as BCAs.

Nevertheless, there are several intriguing aspects of the interaction of insect pests with their BCAs, and the impact of semiochemicals on this interaction is unclear. In the present review, we explore various aspects of the interaction of BCAs with semiochemicals and studies done on the interaction of semiochemicals with insect BCAs (natural enemies and entomopathogenic microbials) (Table 1). We are not including the studies related to the implication of trap crops, where the relevant semiochemicals are not identified.

## 2. Semiochemicals and Natural Enemies (Parasitoids and Predators)

### 2.1. Parasitoids

Semiochemicals play a crucial role in the host–parasitoid relationship in habitat location, host location, host acceptance, and oviposition. Both host plants and host insects play roles in attracting the parasitoids [12]. Non-host plants can also release stimuli influencing parasitoid searching, parasitism, or retention of foragers. Both damaged and undamaged plants attract parasitoids. Allelochemicals from non-host plants attractive to natural enemies can be used as trap crops and combined with deterrent chemical stimuli as an effective means of biological control. Similarly, flowering plant strips close to the main crop increases the biodiversity of beneficial insects by attracting them through the emission of semiochemicals [15,54]. Lewis et al. [55] explained the role of kairomones in the host-finding activities of female parasitic insects. More recently, Murali-Baskaran et al. [15] explained the source of kairomones and their role in host plant–herbivore–entomophage interactions under field conditions. A mini-review cited a list of kairomones and synomones used by insect parasitoids for habitat and host location [56], and also mentioned that the maximum known chemical cues are those for habitat-location, followed by host acceptance and host-location.

The interaction of host’s pheromones and parasitoids has been explored by various researchers, confirming the attraction of parasitoids to the whole-body extract of specific phytophagous insects such as *Corcyra cephalonica* (Stainton) (Lepidoptera: Pyralidae). Extracts of bodies of female *C. cephalonica* were found to be more attractive than males to *Trichogramma chilonis* (Ishii) (Hymenoptera: Trichogrammatidae) and *Chrysoperla zastrowi* sillemi (Esben-Peterson) (Neuroptera: Chrysopidae), due to the presence of large amounts of attractive hydrocarbons, such as tricosane [57]. The hydrocarbons heneicosane and hexacosane, isolated from the whole-body extract of females of *Spodoptera exigua* (Hübner) (Lepidoptera: Noctuidae) and *Chilo auricilius* (Dudgeon) (Lepidoptera: Crambidae), behave as kairomones and create a significant attraction for the parasitoid *T. chilonis*. Similarly, higher levels of docosane and heneicosane, again from female whole-body extracts of *Spodoptera litura* (Fabricius) (Lepidoptera: Noctuidae), attracted more *T. chilonis*, while whole-body extract of *Earias vittella* (Fabricius) (Lepidoptera: Noctuidae), which contained heneicosane, tricosane, pentacosane, hexacosane, octacosane, and nonacosane, was also attractive to *T. chilonis* [15]. Similarly, terpenoids (e.g., the monoterpene (*E*)-β-ocimene and the monoterpene alcohol linalool, the methylene monoterpene (3)-4,8-dimethyl-1,3,7-nonatriene, the methylene sesquiterpene (3*E*,7*E*)-4,8,12-dimethyl-1,3,7,11-tridecatetraene, and the sesquiterpene (*E*)-β-caryophyllene) showed high variability among plant genotypes and played a major role in increasing attractiveness to the parasitoids [58].

Kairomones, which are long-chain hydrocarbons, ketones of fatty acids, esterified cholesterols, or proteins, emitted by host frass or glue used in oviposition, are known as external kairomones. Meanwhile, the kairomones secreted from the host body indicate the suitability of the host for parasitoid progeny and are represented by amino acids and salts in the hemolymph [12,56]. These kairomones can be used in insect pest management, for instance, when wheat plants were sprayed with (*Z*)-jasmone (a compound released from cotton leaves and flowers when fed on by various lepidopteran larvae), they became less attractive to aphids and instead attracted more parasitoids in both laboratory and field conditions, and, due to increased parasitism, the aphid population declined [20]. Insects also produce pheromones when stimulated by plant volatiles; for instance, some female moths deposit pheromones on the host plant and these pheromones attract male moths [15]. The (*E*)-β-farnesene synthase gene (alarm pheromone released by aphids) that is expressed in *Arabidopsis thaliana* (L.) Heynh. (Brassicales: Brassicaceae) causes the emission of (*E*)-β-farnesene, which repels aphids but attracts the parasitoid *Diaeretiella rapae* (M’Intosh) (Hymenoptera: Ichneumonidae) [59]. The scales, silk, and frass of larvae and adults of *Opisina arenosella* (Walker) (Lepidoptera: Crytophasidae) elicited attraction from several parasitoid species, such as *Trichogramma evanescens* (Westwood) (Hymenoptera: Trichogrammatidae), *Goniozus nephantidis* (Muesebeck) (Hymenoptera: Bethylidae), *Brachymeria nephantidis* (Gahan) (Hymenoptera: Chalcididae), and *Elasmus nephantidis* (Rohwer) (Hymenoptera: Eulophidae) [55,60].

Plant pathogens also create complex tritrophic relationships between host plants and phytophagous insects and their parasitoids. Pathogens on plants manipulate a host plant to produce volatiles that attract their vector and also parasitoids [15]. *Candidatus Liberibacter asiaticus* (Jagoueix et al.) (Rhizobiales: Phyllobacteriaceae), for instance, induces citrus trees to release methyl salicylate, which attracts its vector, *Diaphorina citri* (Kuwayama) (Hemiptera: Liviidiae) and also a parasitoid of nymphs of *D. citri*, *Tamarixia radiata* (Waterston) (Hymenoptera: Eulophidae) [15]. Alkaloids, terpenes, flavonoids, and phenolic compounds in flowers of the weed, *Hyptis suaveolens* ([L.] Poit.) (Lamiales: Lamiaceae) in the rice ecosystem were reported to be attractive to *Tetrastichus schoenobii* (Ferriere) (Hymenoptera: Eulophidae), an egg parasitoid of *Scirpophaga incertulas* (Walker) (Lepidoptera: Pyraustidae) [61]. The extracts of flower buds, flowers, and leaves of *Tagetes erecta* (L.) (Asterales: Asteraceae) attract *Helicoverpa armigera* (Hübner) (Lepidoptera: Noctuidae) and its parasitoids, due to the presence of compounds containing benzaldehyde, (*S*)-(–)-limonene, (*R*,*S*)-(±)-linalool, (*E*)-myroxide, (*Z*)-b-ocimene, phenylacetaldehyde, and (*R*)-(–)-piperitone [62]. Interestingly, the combination of kairomones with molasses increased the parasitism of *Agrotis ipsilon* (Hufnagel) (Lepidoptera: Noctuidae) by *Meteorus rubens* (Nees Von Esenbeck) (Hymenoptera: Braconidae) [15,63]. Similarly, volatiles of symbiotic fungus (*Cerrena unicolor* (Bull.) Murrill) which is deposited by wood wasps [*Tremex apicalis* Matsumura (Hymenoptera: Siricidae: Tremecinae)], and consumed as a food source by immatures of wasps, attract *T. apicalis* females, and also attract females of their parasitoids *Ibalia* (*Tremibalia*) *japonica* Matsumura (Hymenoptera: Ibaliidae) [64].

Other semiochemicals, such as herbivore-induced plant volatiles (HIPVs), play a major role in tritrophic interactions and enable parasitoids to recognize their hosts. Specialized and generalist parasitoids distinguish HIPV cues differently. Different volatile blends work together and affect the attractiveness of a particular combination [23,65]. At present, the biosynthesis of many major HIPV classes is known and the genes and proteins involved have been explored. For instance, green leaf volatiles (GLVs) are produced by the oxygenation of fatty acids of plastid membranes through an enzymatic cascade involving lipoxygenases and a hydroperoxide lyase that converts the alcohol into the acetate (*Z*)-3-hexenyl acetate. This final acetate compound is attractive to natural enemies and its emission is regulated by a variety of internal and external factors and can be controlled by plants [66,67,68].

However, the genetic diversity of parasitoid populations and phenotypic plasticity of individuals, together with their physiological state, often results in substantial variations in the response to chemical cues [69]. Kairomones can increase the effectiveness of biological management by increasing predation or parasitism rates. As such, they can be applied to plants to increase the rate of parasitization of pest insects [12,19]. The synergistic relationship between insect pheromones and plant odors can also increase the attraction of natural enemies [8]. The systematic scope of the parasitoid groups could also throw some light on the semiochemical–parasitoid–host insect–host plant relationship. Currently, most examples are of species of braconids, while their host insects mainly belong to the Lepidoptera, followed by Hemiptera, Coleoptera, and Diptera [56].

### 2.2. Predators

Tritrophic relationships among pest insects, their host plants, and the predators of such pests have also been well explored, and plant volatiles have been shown to play a major role in mediating these relationships. When using predators, augmentation biological control can be a viable pest management technique in enclosed environments, but in field conditions, due to the rapid dispersal of predators, this technique is less effective. In contrast to herbivores (which are more sensitive to constitutive host plant volatiles, e.g., green leaf volatiles), both generalist and specialist predators are more sensitive to systemic volatiles produced by the plants in response to the prey feeding [70]. Various predators in the same ecosystem can interact synergistically, additively, or antagonistically. However, there are instances where predators, although present in the same ecosystem, do not interact, for example the aphid-feeding predators *Eriopis connexa* (Germar) and *Hippodamia variegata* (Goeze) (both Coleoptera: Coccinellidae), and *Trirammatus striatula* (Fabricius) (Coleoptera: Carabidae) did not interact [71].

In a study done in Indiana, USA [39], to manage hornworm caterpillars, *Manduca sexta* (L.) (Lepidoptera: Sphingidae), the generalist predatory stink bug *Podisus maculiventris* (Say) (Hemiptera: Pentatomidae) was combined with behavior-modifying semiochemicals, the latter being used to increase the retention of mass-released sting bugs. A formulation of a mixture of the stink bug’s aggregation pheromone (7.6% (*E*)-2-hexenal, 0.4% benzyl alcohol and 92% a-terpineol) with methyl salicylate (MeSA) (an herbivore-induced plant volatile that is attractive to several natural enemy taxa) was used. Both these semiochemicals increased the attack rate on hornworm caterpillars by *P. maculiventris* [29].

It has also been shown that semiochemicals play a role in the interspecific avoidance among a guild of natural enemies attacking the same host in the same habitat. For example, some parasitoids of aphids with varying host ranges (*Aphidius eadyi* [Stary, González and Hall] [Hymenoptera: Aphidiidae]; *Aphidius ervi* Haliday and *Praon volucre* Haliday [both Hymenoptera: Braconidae]) avoided the predators *Coccinella septempunctata* (L.) and *Adalia bipunctata* (L.) (both Coleoptera: Coccinellidae). This avoidance was achieved because the chemical trails of these predators (hydrocarbons n-tricosane [C23H48], n-pentacosane [C25H52], and n-heptacosane [C27H56]) elicited avoidance responses in all three parasitoids [72]. On the other hand, pheromones secreted by the predators are also reported to attract parasitoids [73].

In some cases, the same semiochemical can be used by both an insect pest and its predators in an interaction. Both the bark beetle *Ips pini* (Say) (Coleoptera: Scotylidae) and its predator *Thanasimus dubius* (F.) (Coleoptera: Cleridae) respond to ipsdienol, it being an aggregation pheromone for the bark beetle and a kairomone for prey location for the predator. However, the two species differ in their preferences among enantiomeric blends of ipsdienol, which helps explain the predator–prey coevolution and also the development of resistance by *I. pini* to management strategies [24]. In another study, it was shown that the preferences of *I. pini* and its predators (*Temnochila chlorodia* [Mannerheim] [Coleoptera: Trogossitidae] and *Enoclerus lecontei* [Wolcott] [Coleoptera: Cleridae]) for bark beetle pheromones (ipsdienol and lanierone) varied and showed both an antagonistic and synergistic relationship. Hence, to improve monitoring programs at the regional scale before deploying any semiochemical traps, the type of such interactions should be considered [27]. The use of synthetic pheromone lures to monitor bark beetles and their predators in forest ecosystems are common, nevertheless, attraction varies between natural and synthetic pheromones. Furthermore, other ecological factors such as seasonal flight patterns, and variations in the phenology of phytophagous insects and their natural enemies also play an important role in the success of these synthetic pheromone traps. Before deploying traps in any long-term program, these factors should be considered, and infested host material should also be evaluated before developing estimates of preferred pheromone blends for pest and predator densities [25].

Similar to parasitoids, the frass of prey insects also plays a role in attracting predators, as shown in the case of the bark beetle *Dendroctonus micans* (Kugelann) (Coleoptera: Curculionidae), whose predator *Rhizophagus grandis* (Gyllenhall) (Coleoptera: Rhizophagidae) is attracted to the bark beetle’s frass, present in the tree [26]. In another study, it was shown that bark decay also plays a role in the interaction of prey and predators. The spruce bark beetle, *Ips typographus* (L.) (Coleoptera: Curculionidae), and its predators *Medetera setiventris* (Thuneberg) (Diptera: Dolichopodidae), *Thanasimus formicarius* (L.) and *Thanasimus femoralis* (Zetterstedt) (both Coleoptera: Cleridae) were tested for various compounds secreted by *I. typographus*, and different stages of tree decay influence the development of the bark beetle [28].

## 3. Semiochemicals and Entomopathogenic Microbials (Fungi, Nematodes, Bacteria, Viruses)

### 3.1. Fungi

Pathogens may be dispersed naturally by parasitoids, predators, and the feces of insects, birds, and mammals, and surface contamination [18]. However, for entomopathogenic fungi, natural dispersal, in additional to the aerial movement of spores, is also known to occur through the movement of the targeted insect pests and pollinators, as shown in honey bees in canola production, where honey bees disperse *B. bassiana*, increasing the mortality of *Lygus* sp. (Hahn) (Hemiptera: Miridae) [74,75]. A selective and assisted dissemination technique called auto-dissemination is also extremely helpful in spreading entomopathogens [18]. Auto-dissemination can be used to target both adults and larvae of some insect pests [3,13,76]. Semiochemicals are being used to increase the rates of fungal infection in several insects. Successful examples of the use of combinations of semiochemicals and entomopathogenic fungi include bark beetles (*I. typographus*), weevils (*Cylas formicarius* Fabricius [Coleoptera: Brentidae], *Cosmopolites sordidus* Germar [Coleoptera: Curculionidae]), moths (*Plutella xylostella* L. [Lepidoptera: Plutellidae]), stink bugs (*Plautia crossota* stali Scot [Hemiptera: Pentatomidae]), thrips (*Megalurothrips sjostedti* Trybom [Thysanoptera: Thripidae]), and aphids (*Phorodon humuli* Schrank [Hemiptera: Aphididae]) [16]. To make this method successful, an appropriate physical separation (including the distance) between semiochemicals and entomopathogenic fungus is needed to achieve the maximum output of autoinoculation [43].

Plants also host entomopathogenic fungi naturally [77], that remain as endophytic fungi after the conidia of an entomopathogenic fungus germinate and enter the plant cuticle [78]. The presence of these endophytic entomopathogenic fungi in plants causes mycosis in different insect pests [79]. Epiphytic fungi on plants are also reported to attract insects. Western yellowjacket [*Vespula pensylvanica* Saussure (Hymenoptera: Vespidae)] and the German yellowjacket [*V. germanica* Fabricius (Hymenoptera: Vespidae)] vector the fungus *Aureobasidium pullulans* ([de Bary] Arnaud) (Dothideales: Dothioraceae). A study done in orchards in Washington, USA found that the volatile compounds emitted by this fungus can attract eusocial wasps and that wasps and fungi appear to have a symbiotic relationship [80]. In a laboratory experiment also done in the USA, it was found that the hymenopteran parasitoids *Roptrocerus xylophagorum* (Ratzeburg) (Hymenoptera: Pteromalidae) and *Spathius pallidus* (Ashmead) (Hymenoptera: Braconidae) are attracted to bluestain fungi (genus *Ophiostoma* [Syd. and P. Syd.]), which are associated with bark beetles (Coleoptera: Scolytidae) feeding in pine trees. This study also found that such fungus-based attraction might not function for short-range host location [81].

The ‘lure and kill’ method has been highly effective for controlling some insect pests by using semiochemicals (especially pheromones) in conjunction with entomopathogenic fungi. Successful examples include the management of sap-sucking insects such as aphids (*P. humuli*), thrips (*M*. *sjostedti*), green bugs (*P. crossota*), and chewing and biting insect pests such as bark beetles (*I. typographus*), weevils (*C. formicarius* and *C. sordidus*), and moths (*P. xylostella*) [16]. Nevertheless, in most cases, such as sex-specific semiochemicals, which attract only one sex, the method is less effective. In addition, the ‘lure and kill’ method is still not well developed for soil-dwelling insects, although Agriculture and Agri-Food Canada (AAFC) (Agassiz, BC, Canada) has created prototype granules of *Metarhizium brunneum* (Petch) (Hypocreales: Clavicipitaceae) combined with pheromone compounds that have showed some promising results for attracting species of *Agriotes* cutworms (Coleoptera: Elaterideae) to bait sources [42]. The use of pheromones in granulated or in pellet form could work well for soil-dwelling insects [13].

### 3.2. Nematodes

The efficacy of entomopathogenic nematodes (EPNs) mainly depends on the strain, formulation, and method of application [82]. However, in recent studies, several HIPVs from the roots of host plants that attract EPNs were examined as formulation additives. These HIPVs are secreted at damaged sites when their production is triggered by compounds in the saliva of phytophagous insects during feeding. Plants also release defense-related volatiles that can attract EPNs [83,84,85]. Furthermore, volatiles secreted by such nematodes also attract EPNs; for instance, the application of infected cadavers with EPNs proved to be more effective than the direct spraying of infective juveniles. When an extract of the infected cadavers was applied along with the aqueous suspension of *Heterorhabditis bacteriophora* (Poinar) Hb strain (Rhabdita: Heterorhabditidae), it was also found to be more infective than direct spraying to *Galleria mellonella* (L.) (Lepidoptera: Pyralidae) [44]. In another study, macerated hosts infected with *Steinernema carpocapsae* (Weiser) All strain, *Steinernema feltiae* (Filipjev) SN strain (both Rhabdita: Steinernematidae) and *H. bacteriophora*, increased the dispersal of these EPNs in soil columns [46]. Ascarosides (a group of glycolipids which regulate mating and development) secreted by several EPN species result in a greater dispersal of various EPNs, in both natural and synthetic form [83]. Pheromone extracts from *S. feltiae* (SN strain) or *S. carpocapsae* (All strain), when tested on *Tenebrio molitor* (L.) (Coleoptera: Tenebrionidae) larvae, showed an improved dispersal and efficacy, which suggests that pheromone-mediated enhancement of EPN efficacy could be achieved by exposing EPNs to specific pheromones [47].

### 3.3. Viruses

Most known entomopathogenic viruses are baculoviruses (four genera: Alpha-, Beta-, Gamma-, and Deltabaculoviruses), Reoviridae, Parvoviridae, or Nudiviruses [4]. The use of semiochemicals for the dispersal of entomopathogenic viruses has not been studied extensively. However, a combination of apple-associated yeasts and codling moth granulovirus (CpGV) increased the mortality of the codling moth (*Cydia pomonella* (L.) (Lepidopetra: Tortricidae) under both laboratory and field conditions [86]. The pheromone is known to increase the efficiency of the Granuloviruses in the insect pests *C. pomonella* and *Adoxophyes orana* (Fischer von Röslerstamm) (Lepidopetra: Tortricidae) [49]. In 1992, the potential of sex pheromone baited traps was first evaluated [48] in the USA, to auto-disseminate the *Baculoviruses* [nucleopolyhedrovirus (NPV)] against *Heliothis virescens* (Fabricius) (Lepidoptera: Noctuidae) [18,48].

### 3.4. Bacteria

Among entomopathogenic bacteria, the best known is *Bacillus thuringiensis.* It has been known since 1901 and is used to manage several major insect pests in agriculture, forestry, and medicine [4]. Although the use of autoinoculator devices is reported to aid in dispersal of *Paenibacillus* (=*Bacillus*) *popilliae* (Dutky) (Eubacteriales: Bacillace) to manage *Popillia japonica* Newman (Coleoptera: Scarabaediae) [18,87], the use of semiochemicals to improve the efficacy and dispersal of bacteria has not been explored.

### 3.5. Protozoa

The inclusion of semiochemicals in the dispersion of protozoans to manage insect pests is a scantly explored area and needs further exploration. Shapas et al. [53] evaluated generations of *Trogoderma glabrum* (Herbst) and indicated that they were reduced after the dispersal of protozoan pathogen spores, *Mattesia trogodermae* Canning. Pheromone-baited (synthetic sex pheromone, (*E*)-14-methyl-8-hexadecenal) spore-transfer sites were used to disperse the spores. In this study, it was also indicated that males became attracted to females and these males induced attempted copulation with the pheromone source, aiding in spore transfer to males [53].

## 4. Future Perspective and Advancements

The interaction of semiochemicals with biocontrol agents is multidimensional. Although much knowledge regarding pheromones and plant-based semiochemicals is available for different insect pests, the interaction of semiochemicals with biocontrol agents, especially microbials, is a less explored area. Similarly, our understanding of the ecology and evolution of semiochemicals relative to biocontrol agents is at a very early stage and requires further study.

Complete chemical profiles of the plants hosting natural enemies, and exploration of the genotype within the species, both need to be better determined to explore this tri-trophic interaction. Estimating the optimal release rates of synthetic semiochemicals can increase the foraging efficiency of insect biocontrol agents. Further exploration of non-host plants for possible use as trap crops and intercrops is needed to improve the conservation use of biological control agents. Identification of kairomones from whole-body washes of insect pests could play a major role in future biological control programs. Exploration of the source cues of kairomones in both laboratory and field experiments is needed to integrate more kairomones with biocontrol agents. An improved understanding of the genes responsible for the production of semiochemicals will certainly help with this. Also needed are more field studies focusing on the proper dosage and economic analyses [15,88]. Studies of the insect pests for which the chemical composition of various semiochemicals is already known should be focused on integrating the known chemical compounds with microbial biocontrol agents.

The most prevalent tri-trophic interaction has lately been reclassified as a multi-trophic interaction, since several other ecological factors play a major role in these interactions [89]. Further exploration of the interaction of various BCAs with each other, and the compatibility between them, can ensure greater success. The role of symbionts (in insects) and endophytes (in plants) on BCAs, and the possibility of manipulating their emission of semiochemicals, also needs further exploration. The incorporation of known semiochemicals to attract natural enemies under field conditions is increasing in prevalence. Nevertheless, information about the precise identity of attractive compounds, their amount, the method of application, and their release rate are still often unknown. Other than the identification of semiochemicals, the application of identified compounds to attract natural enemies involves a precise and meticulous process to develop an effective biological control program [12,18].

The manipulation of population levels of natural enemies by semiochemicals involves a chemical application, habitat manipulation, host plant manipulation, and parasitoid manipulation. Both conserving and recruiting natural enemies are part of creating a suitable ecological infrastructure. For natural enemies, the selection of appropriate HIPVs, floral odors, and host-associated cues, their testing in laboratory and field conditions, combining them with other biological control methods, and, finally, the regular monitoring of natural enemies and their activity, density, parasitism rate, and also the density of the host, host damage, and yields, are needed for successful implementation. Moreover, the direct application of semiochemicals in the field involves the application of synthetic compounds, individually or in the mixture through slow-release dispensers, to act directly on natural enemies, or the application of synthetic compounds to induce plant’s chemical defense to attract natural enemies. For natural enemies, the ‘attract and reward’ strategy is also used, which helps in retaining the natural enemies by implementing the food source as traps [90].

The entire process involves laboratory bioassays, optimizing semiochemical-baited traps, the preparation and calibration of traps, and determining the extent of attraction in field conditions [12]. For the dispersion of microbials, auto-dissemination is becoming a major area of study for efficient and cost-effective use of these microbial biological control agents, especially in the context of ‘lure and kill’ systems. Trap deployment, equipment, and application strategy for auto dissemination are critical aspects of creating a successful pest management system [18]. Lure and infect strategies and optimization of the devices is the biggest challenge for the incorporation of semiochemicals with microbials [16].

The number of studies conducted in field conditions using semiochemicals has increased in recent years, but information is still scarce. Considering these issues, one alternative may be to manipulate the plants through breeding or genetic engineering to produce and release specific volatiles [91].

## 5. Conclusions

The use of semiochemicals and their inclusion with biological control agents has increased in the recent decades, especially for auto-dissemination of microbials and the incorporation of trap and cover crops to attract natural enemies and increase their biodiversity. Semiochemicals affecting parasitoids and predators are fairly well studied, but knowledge of semiochemicals affecting microbial biological control agents is still sparse, and more work is needed to develop effective application strategies to incorporate semiochemicals with the BCA. Chemical communication compounds are known for several important insect pests; further work should be focused on the groups of insects for whom the semiochemicals are best known. The following points should be addressed in future studies: determining the type of relationship between known semiochemicals and BCA; exploration of the incorporation of semiochemicals with entomopathogenic virus and bacteria; exploration of more effective formulations of microbials (for example, granular formulations of EPFs and bio-degradable trap material) and their incorporation with semiochemicals. Finally, species-specific tactics may be needed where crops have pest complexes that need control.

## Figures and Tables

**Table 1:** Examples of implication of semiochemicals enabling greater efficacy of biological control agents.

	Biological Control Agent	Insect Pest	Host Plant	Type of Semiochemical	Reference
**1.**	**Parasitoids**				
i.	*Trichogramma* (Riley) spp. (Hymenoptera: Trichogrammatidae)	*Heliothis zea* (Boddie) (Lepidoptera: Noctuidae) and *Anticarsia gemmatalis* Hübner (Lepidoptera: Noctuidae)	*Glycine max* ((L.) Merr.) and *Trifolium incarnatum* (L.) (both Fabales: Fabaceae)	synthetic tricosane	[19]
ii.	*Aphidius ervi* (Haliday) (Hymenoptera: Braconidae)	*Rhopalosiphum padi* (Linnaeus) (Hemiptera: Aphididae)	*Vicia faba* (L.) (Fabales: Fabaceae)	cis-Jasmone	[20]
iii.	*Oomyzus gallerucae* (Fonscolombe) (Hymenoptera: Eulophidae))	*Xanthogaleruca luteola* Müller (Coleoptera: Chrysomelidae)	*Ulmus minor* (Mill.) (Rosales: Ulmaceae)	Terpenoids	[21]
iv.	*Trissolcus* (Ashmead) spp. (Hymenoptera: Platygastridae)	*Euschistus heros* (Fabricius) (Hemiptera: Pentatomidae)	*Glycine max*	(*E*)-2-hexenal	[22]
v.	*Telenomus podisi* (Ashmead) (Hymenoptera: Platygastridae), *Trisscolus teretis* (Johnson (Hymenoptera: Scelionidae)	*Euschistus heros* fabricius (Hemiptera: Pentatomidae)	resistant *Glycine max* cultivars Dowling and IAC 100	(*E*,*E*)-α-farnesene, methyl salicylate, (*Z*)-3-hexenyl acetate, and (*E*)-2-octen-1-ol	[23]
**2.**	**Predators**				
i.	*Thanasimus dubius* (Fabricius) (Coleoptera: Cleridae)	*Ips pini* (Say) (Coleoptera: Curculionidae)	*Pinus strobus* (L.) (Pinales: Pinaceae)	Ipsdienol	[24,25]
ii.	*Rhizophagus grandis* (Gyllenhall) (Coleoptera: Rhizophagidae)	*Dendroctonus micans* (Kugelann) (Coleoptera: Curculionidae)	-	monoterpenes and oxygenated monoterpenes	[26]
iii.	*Coccinella septempunctata* (L.) (Coleoptera: Coccinellidae)	*Rhopalosiphum* *padi*	*Vicia faba*	cis-Jasmone	[20]
iv.	*Temnochila chlorodia* (Mannerheim) (Coleoptera: Trogossitidae) and *Enoclerus lecontei* (Wolcott) (Coleoptera: Cleridae)	*Ips pini*	*Pinus strobus*	ipsdienol and lanierone	[27]
v.	*Medetera setiventris* (Thuneberg) (Diptera: Dolichopodidae), *Thanasimus formicarius* (L.) (Coleoptera: Cleridae) and *Thanasimus femoralis*	*Ips typographus* (L.) (Coleoptera: Curculionidae)	*Picea abies* ([L.] H. Karst.) (Pinales: Pinaceae)	*S*-cis-verbenol, 2-methyl-3-buten-2-ol, ipsdienol, (+)-a-pinene, (–))-a-pinene, (±)-a-pinene, limonene, Camphor and ipsdienol	[28]
vi.	*Podisus maculiventris* (Say) (Coleoptera: Pentatomidae)	*Manduca sexta* (L.) (Lepidoptera: Sphingidae)	*Solanum lycopersicum* (L.) (Solanales: Solanaceae)	Methyl salicylate (MeSA), or *Podisus maculiventris* aggregation pheromone	[29]
**3.**	**Entomopathogenic Fungus**				
1.	*Trichothecium roseum* ([Pers.] Link) (Hypocreales: Incertae sedis)	*Oryzaephilus surinamensis* (L.) and *O. mercator* (Fauvel) (both Coleoptera: Silvanidae) *Cryptolestes ferrugineus* (Stephens) (Coleoptera: Laemophloeidae), *Ahasverus advena* (Waltl) (Coleoptera: Silvanidae), *Cathartus quadricollis* (Guerin-Meneville) (Coleoptera: Silvanidae)	-	l-Octen-3-one, racemic 3-octanol, and 3-octanone	[30]
ii.	*Zoophthora radicans* (Brefeld) (Zygomycetes: Entomophthorales)	*Plutella xylostella* (L.) (Lepidoptera: Yponomeutidae)	*Brassica chinensis* (L.) var. *pekinensis* (Rupr.) Sun. (Brassicales: Brassicaceae)	(*Z*)-ll-hexadecenal, (*Z*)-ll-hexadecenyl acetate, (*Z*)-ll-hexadecanol and (Xg BHT antioxidant (di-tertbutyl-4-methylphenol) in hexane	[31,32]
iii.	*Beauveria bassiana* (Bals.-Criv.) Vuill. (Hypocreales: Cordycipitaceae)	*Cylas formicarius* (Fabricius) (Coleoptera: Brentidae)	-	-	[33]
Iv.	*Verticillium lecanii* ([Zimm.] Viégas) (Hypocreales: Cordycipitaceae)	*Phorodon humuli* (Schrank) (Hemiptera: Aphididae)	*Prunus domestica* (L.) (Rosales: Rosaceae)	nepetalactol	[34]
v.	*Beauveria bassiana*	*Plautia crossota* stali (Scot) (Hemiptera: Pentatomidae)	Orchards	Aggregation pheromone	[35]
vi.	*Zoophthora radicans*	*Plutella xylostella*	*Brassica oleracea* (L.) (Brassicales: Brassicaceae)	Synthetic sex pheromone (1: 1 mix of (*Z*)-11-hexadecenal and (*Z*)-11-hexadecenyl acetate)	[36]
vii.	*Beauveria bassiana*	*Ips typographus*			[37]
viii.	*Beauveria bassiana*	*Cosmopolites sordidus* (Germar) (Coleoptera: Curculionidae)	-	aggregation pheromone sordidin (Cosmolure^®^)	[38,39]
ix.	*Metarhizium anisopliae* ([Metchnikoff] Sorokin) (Hypocreales: Clavicipitaceae)	*Amblyomma variegatum* (Fabricius) (Ixodida: Ixodidae)	-	attraction-aggregation-attachment pheromone (AAAP), made up of o-nitrophenol, methyl salicylate and nonanoic acid in the ratio 2:1:8, 1-octen-3-ol and butyric acid with CO2	[40,41]
x.	*Metarhizium anisopliae*	*Frankliniella occidentalis* (Pergande) (Thysanoptera: Thripidae)	*Phaseolus vulgaris* (L.) var. Samantha (Fabales: Fabaceae).	Lurem-TR	[17]
xi.	*Metarhizium brunneum* (Petch) (Hypocreales: Clavicipitaceae)	*Agriotes obscurus* (L.) (Coleoptera: Elateridae)	-	(1 % wt/wt 1:1 geranyl hexanoate:geranyl octanoate	[42]
xii.	*Metarhizium brunneum* and *Metarhizium anisopliae*	*Megalurothrips sjostedti* (Trybom) (Thysanoptera: Thripidae)	*Vigna unguiculata* ([L.] Walp.) (Fabales: Fabaceae)	Lurem-TR, a commercial semiochemical (active ingredient is methyl-isonicotinate)	[43]
**4.**	**Entomopathogenic Nematodes**				
i.	*Heterorhabditis bacteriophora* (Poinar) (Rhabdita: Heterorhabditidae)	*Galleria mellonella* (L.) (Lepidoptera: Pyralidae)	-	-	[44]
ii.	*Steinernema feltiae* (Filipjev) (Rhabdita: Steinernematidae)	*Galleria mellonella*	-	-	[45]
iii.	*Steinernema carpocapsae* (Weiser) (Rhabdita: Steinernematidae), *Steinernema feltiae*, *Heterorhabditis bacteriophora*	*Galleria mellonella*	-	-	[46]
iv.	*Steinernema feltiae* and S *Steinernema carpocapsae*	*Tenebrio molitor* (L.) (Coleoptera: Tenebrionidae)	-	-	[47]
**5.**	**Entomopathogenic Virus**				
i.	Baculoviruses [nucleopolyhedrovirus (NPV) *Autographa californica* nuclear polyhedrosis virus (AcNPV) (Baculoviridae)	*Heliothis virescens* (Fabricius) (Lepidoptera: Noctuidae)	-	-	[18,48] (references cited in Vega et al. [18])
ii.	Baculoviruses (BV) Summer fruit totrix GV(AdorGV)	*Cydia pomonella* (L.) (Lepidopetra: Tortricidae), *Adoxophyes orana* (Fischer von Röslerstamm) (Lepidopetra: Tortricidae)	-	-	[49,50,51,52]
**6.**	**Protozoa**				
i.	*Mattesia trogodermae* (Canning) (*Neogregarinorida*) Lipotrophidae	*Trogoderma glabrum* (Herbst) (Coleoptera: Dermestidae)	-	synthetic sex pheromone, (*E*)-14-methyl-8-hexadecenal	[53]
